# Deep Brain Stimulation: A Paradigm Shifting Approach to Treat Parkinson's Disease

**DOI:** 10.3389/fnins.2016.00173

**Published:** 2016-04-28

**Authors:** Patrick Hickey, Mark Stacy

**Affiliations:** Department of Neurology, Duke University Medical CenterDurham, NC, USA

**Keywords:** Parkinson's disease, deep brain stimulation, subthalamic nucleus, globus pallidus, pedunculopontine nucleus

## Abstract

Parkinson disease (PD) is a chronic and progressive movement disorder classically characterized by slowed voluntary movements, resting tremor, muscle rigidity, and impaired gait and balance. Medical treatment is highly successful early on, though the majority of people experience significant complications in later stages. In advanced PD, when medications no longer adequately control motor symptoms, deep brain stimulation (DBS) offers a powerful therapeutic alternative. DBS involves the surgical implantation of one or more electrodes into specific areas of the brain, which modulate or disrupt abnormal patterns of neural signaling within the targeted region. Outcomes are often dramatic following DBS, with improvements in motor function and reductions motor complications having been repeatedly demonstrated. Given such robust responses, emerging indications for DBS are being investigated. In parallel with expansions of therapeutic scope, advancements within the areas of neurosurgical technique and the precision of stimulation delivery have recently broadened as well. This review focuses on the revolutionary addition of DBS to the therapeutic armamentarium for PD, and summarizes the technological advancements in the areas of neuroimaging and biomedical engineering intended to improve targeting, programming, and overall management.

## Introduction

Parkinson disease (PD) is a chronic neurodegenerative disorder classically characterized by motor manifestations including slowed voluntary movements, resting tremor, muscle rigidity, and impaired gait and balance (Stacy, 2009). With a prevalence of 0.3% that increases with advancing age (Fahn, 2003), PD is projected to have a significant worldwide economic impact in the coming years (Dorsey et al., 2007; Kowal et al., 2013). Although the pathogenesis of PD has not been fully elucidated, motor deficits are associated with selective loss of pigmented neurons, which originate in the substantia nigra pars compacta and terminate in the striatum (Jellinger, 2007). Medical therapies are largely aimed at increasing the availability of striatal dopamine and typically result in a brisk symptomatic improvement in most PD patients. Unfortunately, this response becomes less reliable and less predictable over time, and after 5 years of therapy, medication-related complications develop in a majority of patients. Motor complications, such as dyskinesia, wearing-off, and “on–off” fluctuations, characterized by a sudden, sometimes unpredictable loss of treatment effect are well-recognized (Nutt, 2001; Stacy, 2009). Non-motor complications are also prevalent and include impulse control disorders, such as pathological gambling, compulsive spending, binge eating, hyperlibidinous behaviors, and compulsive motor activity (Weintraub et al., 2015).

In advanced stages of PD, when medications no longer adequately control motor symptoms, deep brain stimulation (DBS) offers a powerful therapeutic alternative. DBS is a surgical therapy involving the implantation of one or more electrodes into specific regions of the brain, which deliver electrical stimuli to modulate or disrupt abnormal patterns of neural signaling within the targeted region. The results are often dramatic, with improvements in motor function and motor complications having been repeatedly demonstrated. Given such robust responses, emerging indications for DBS are being investigated. In parallel with expansions of therapeutic scope, advancements within the areas of neurosurgical technique and the precision of stimulation delivery have recently broadened as well. This review summarizes the revolutionary addition of DBS to the therapeutic armamentarium for PD as well as recent advances in treatment delivery.

## Historical perspective of deep brain stimulation

Prior to the discovery of levodopa, surgical interventions were the most efficacious treatment for PD symptoms, but primarily focused on the reduction of bothersome tremor. Early approaches targeted the pyramidal tracts, with lesioning either at the point of origin in the cortex or the descending pathways through the brainstem and cervical spinal cord (Cooper, 1956). Although tremor was reliably improved following surgery, hemiparesis was an inevitable consequence. However, in 1952, Dr. Irving Cooper inadvertently interrupted the anterior choroidal artery while performing a mesencephalic pedunculotomy in a patient with PD. Ligation of the vessel was required, though what resulted was a serendipitous reduction in rigidity and tremor with preservation of motor and sensory function. Cooper reasoned the favorable outcomes were due to infarction of the medial globus pallidus. An expansion of ablative stereotactic surgery followed, aided by the earlier development of the stereotactic frame and methods of targeting deep brain structures, including the basal ganglia and thalamus. However, the success of these approaches was limited, partly because of inaccurate, imprecise, and inconsistent targeting. Moreover, intentionally created bilateral brain lesions frequently led to irreversible deficits in speech, swallowing, and cognition.

Surgical intervention for PD faded in popularity in the early 1970s after the arrival of levodopa, which radically transformed the pharmacological treatment of PD. Levodopa was soon accepted as the preferred treatment for PD while demonstrating a positive impact on disability, quality of life, and longevity. However, it also became clear that patients on levodopa and other antiparkinsonian drugs often develop significant drug-induced complications, such as involuntary movements (dyskinesias), motor fluctuations (wearing-off, and “on–off” fluctuations), hallucinations, and psychosis. Such complications, combined with a growing understanding of basal ganglia and cerebellar circuitry, rekindled interest in earlier surgical procedures.

In 1987, Dr. Alim-Louis Benabid explored high frequency stimulation of the thalamus while operating on an elderly man who had previously undergone contralateral ablation for tremor. Given his concerns for creating bilateral lesions, Benabid implanted a wire into the untreated thalamus with four metal contacts at its tip, which was then connected to an external battery source. By passing high-frequency electrical stimulation through the wire, Benabid and colleagues demonstrated that chronic electrical stimulation of the subcortical brain (DBS) was as effective as thalamotomy for tremor suppression (Benabid et al., 1994). Soon thereafter, the use of DBS at other basal ganglia locations was explored for refractory movement disorders, including PD.

## Clinical experience with deep brain stimulation

The advent of modern DBS led to a major change in the therapeutic armamentarium for movement disorders. DBS rapidly overtook lesioning as the surgical treatment of choice for refractory movement disorders due to a number advantages: it is nondestructive and several stimulation parameters, including the location, size, intensity, and the shape of the stimulating current field can be adjusted following surgical implantation. These properties allow clinicians to program the DBS device in such a way as to maximize motor benefits while minimizing side effects, most of which are caused by the inadvertent stimulation of structures adjacent to the intended target. Perhaps most importantly for patients with PD, DBS has a lower reported complication rate when used bilaterally (Tasker, 1998).

Since the first application of DBS for PD in 1993, several thousand patients worldwide have undergone surgical implantation. While many studies have reported the benefits and durability of this therapy (Krack et al., 2003; Fasano et al., 2010; Moro et al., 2010), six large-scale, randomized, controlled clinical trials have been performed (Deuschl et al., 2006; Okun et al., 2009, 2012; Weaver et al., 2009; Williams et al., 2010; Schuepbach et al., 2013). Given the pervasive nature of this disease, the end points of these trials have appropriately included quality of life measures, the severity of motor symptoms in the medication “off” state, and time spent in the “on” state without troublesome motor symptoms (e.g., dyskinesia, as assessed by means of a dyskinesia diary).

The first of these trials was conducted in Germany and Austria and included 156 PD patients with advanced disease and severe motor symptoms (Deuschl et al., 2006). Subjects were randomly assigned to undergo bilateral DBS of the subthalamic nucleus (STN) or to receive optimized and individualized drug therapy according to best practice guidelines. At 6 months, patients assigned to receive DBS had significantly better quality of life measures (mean PDQ-39 31.8 vs. 40.2) and motor scores (mean UPDRS-III 28.3 vs. 46.0). In addition, dyskinesia in the “off” state reduced by 54% in the DBS group but remained unchanged in the medication group.

**Figure 1:**
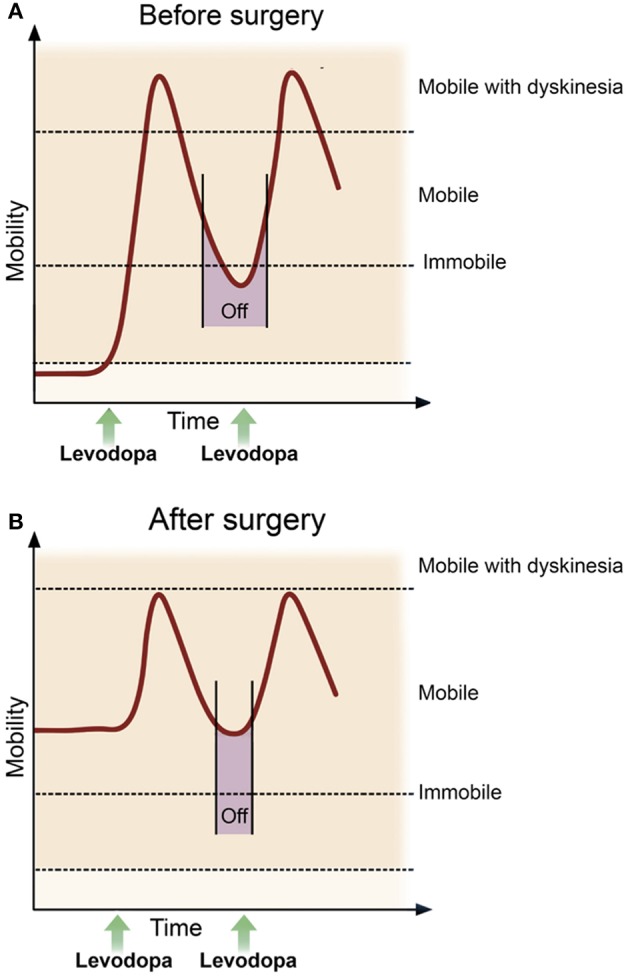
**Mobility and motor function pre- and post-deep brain stimulation**. In advanced Parkinson's disease **(A)** the duration of response after a single dose of levodopa becomes progressively shorter, thus it becomes increasingly difficult to maintain a satisfactory ON state, with OFF periods (purple bar) and dyskinesia often predominating. While most patients continue to take medication after surgery, the goal following DBS **(B)** is a reduction in the duration and severity of OFF time, more consistent and longer ON time, as well as an improvement in dyskinesia. Advances in sensing and stimulating technologies promise to enhance DBS delivery, further reducing variability in motor function and improving clinical efficacy. Adapted from Deuschl and Agid (2013).

Four subsequent trials demonstrated similar efficacy of DBS in advanced PD patients (Okun et al., 2009, 2012; Weaver et al., 2009; Williams et al., 2010). Taken together, the most important benefit of neurostimulation is the improvement of motor function during the “off” state, in particular reducing the duration and severity of motor symptoms when medications are least effective. Thus, DBS patients are likely to experience an improvement in mobility during times when it would otherwise be at its poorest, thereby allowing a more reliable and higher level of overall function. Furthermore, neurostimulation results in a reduction in the subjectively measured “off” time and in the severity of dyskinesias during the “on” state (Figure 1).

At present, DBS is typically considered in advanced PD and thus limited to patients for whom medications are no longer providing adequate relief of symptoms. As a result, patients who undergo DBS are a mean of 58.6 years old with mean disease duration of 12 years (Deuschl et al., 2006; Okun et al., 2009, 2012; Weaver et al., 2009; Williams et al., 2010). In targeting this select patient population, neurostimulation is used in fewer than 2% of all patients with PD. A more widespread and inclusive application of surgery may be restrained by a number of factors, including a lack of familiarity with the procedure, the presence of conditions or comorbidities that may make patients ineligible for the surgery, or concerns for potential surgical risks (especially in elderly individuals) (Deuschl et al., 2013a). The effect that advancing age has on DBS candidacy, surgical complications, and outcomes is of increasing importance as the number of individuals with PD is expected to rise in the coming years (Dorsey et al., 2007). Encouragingly, there is accumulating evidence supporting the safety and efficacy of DBS in elderly PD patients (DeLong et al., 2014; Levi et al., 2015; Mitchell et al., 2015). Together, these reports suggest that age alone should not be a primary exclusion factor for determining surgical candidacy. Instead, all patients should be viewed on the basis of their medical issues, their disease symptoms (i.e., tremor, dyskinesias, gait and postural instability, etc), and whether there is sufficient lifespan to offer a long-term benefit.

While DBS appears to be a practical option in aged individuals, the progressive nature of PD suggests that advanced age will be associated with accumulating impairments in quality of life and limitations of psychosocial function. Given the robust response to neurostimulation in later stages of PD, consideration has been given to whether DBS should be considered earlier in the disease course, perhaps preventing the development of motor complications at a much earlier time point and reducing the burden of such long-term sequelae. To help address this concern, the recently reported EARLYSTIM trial evaluated the benefit of STN DBS compared with medical treatment in 251 PD patients with a mean of 7.5 years of disease duration and at a mean age of 52.5 years (Schuepbach et al., 2013). Subjects were followed for up to 2 years in this multicenter prospective study. Compared with the medical treatment group, outcomes including quality of life and motor disability favored stimulation in these patients just beginning to experience complications of medical therapy. This important investigation provided compelling evidence supporting STN neurostimulation at an earlier disease time point than traditionally considered with regard to cogent motor and non-motor outcome parameters. However, some caution may be advised in extending the promising results to routine clinical practice as patients met strict inclusion criteria with the absence of contraindications, and the study was completed at highly experienced implantation centers under the supervision of multidisciplinary surgical teams (Deuschl and Agid, 2013).

It has been further suggested that DBS at the earliest stage of motor PD may slow the progression of the disease, actually preventing the development of late-stage complications and providing a better overall quality of life (Charles et al., 2008; Hacker et al., 2015). A recent pilot study in PD patients being treated medically for 6 months to 4 years at enrollment, and without motor complications, demonstrated the feasibility of enrolling subjects with early stage PD (Charles et al., 2014; Hacker et al., 2015). After two years of follow up, the surgical group experienced a reduction in the relative risk of symptom worsening (which was defined as both a ≥3 point increase in UPDRS Part III and a ≥1 point increase in UPDRS Part IV) and required less PD medications. Additional long-term analysis will provide further insight into potential benefits of DBS in this cohort, though as with any invasive therapy, safety will also be an important determinant of widespread application in this population (Hariz, 2015).

It is clear that DBS has revolutionized the treatment of advanced PD, providing improvements in quality of life and motor function, while allowing for reductions in drug-related dyskinesia. Extending beyond these measures, evidence suggests that neuromodulation may even reduce mortality and admissions to a residential care home (Ngoga et al., 2014). In addition, recent investigations have further established the safety and efficacy of this therapy at both ends of the disease spectrum, possibly expanding the utilization of this powerful therapy.

## Individualized stimulation delivery

**Figure 2:**
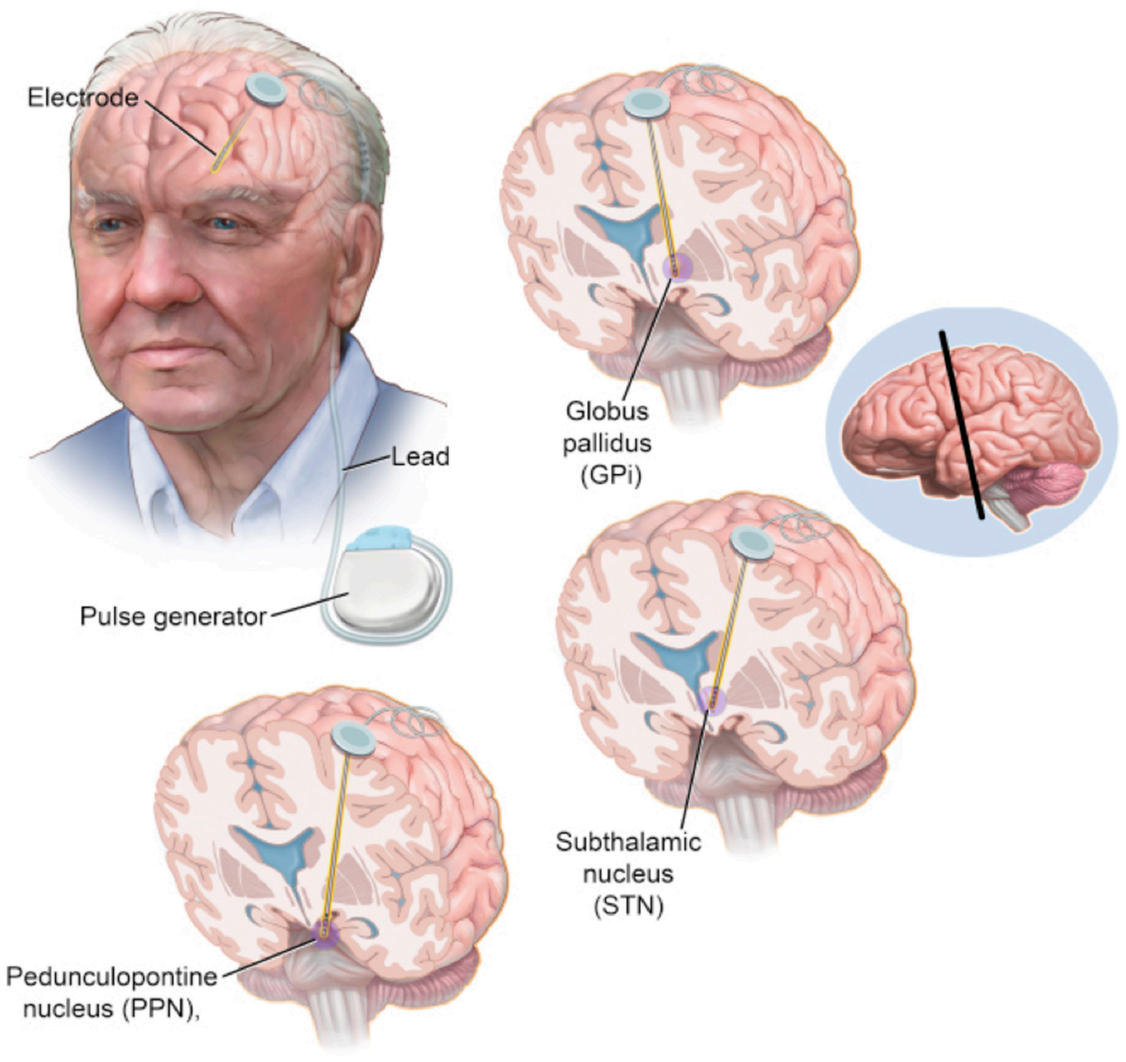
**Electrode Location for Deep Brain Stimulation**. Approved locations for implantation of deep brain stimulation (DBS) include both the subthalamic nucleus and the internal segment of the globus pallidus. The pedunculopontine nucleus has showed promise as a potential DBS target, especially in regards to axial symptoms such as gait and balance dysfunction. During implantation, a burr hole is made in the skull through which the electrode is passed down to the target. An extension is tunneled under the skin of the scalp and connected to an impulse generator located in the chest below the clavicle.

The two most widely utilized and investigated brain targets for PD are the STN and the internal segment of the globus pallidus (GPi; Figure 2). Both have been studied extensively and provide fairly equivalent improvements in the cardinal motor symptoms of PD, dyskinesia, and quality of life. However, some notable differences between these two targets may influence surgical decisions for an individual patient. For example, the Veterans Affairs Cooperative Study showed that STN stimulation had a higher rate of potential worsening of cognition and mood, but allowed more aggressive medication reduction after surgery (Follett et al., 2010). Further, GPi stimulation has been associated with better control of axial motor symptoms including speech and swallowing function (St George et al., 2010; Odekerken et al., 2013; Troche et al., 2014). These distinctions highlight the importance of a comprehensive patient evaluation by a multidisciplinary team in order to maximize potential surgical outcomes.

Unfortunately, interventions at these conventional targets are generally not effective against levodopa-unresponsive symptoms such as gait and balance disturbances and freezing of gait, which are important determinants of quality of life and significantly impact activities of daily living (Table 1). This may be explained by the degeneration of several non-dopaminergic neuronal structures and feedback loops, which likely also contribute to certain PD motor features and appear crucial in the development of gait and balance disorders (Grabli et al., 2013). Thus, novel stimulation techniques, brain targets, and technologies have been investigated in an attempt to expand the utility of DBS and treat symptoms that are less responsive following standard DBS implantation.

**Table 1:** **The Effect of Levodopa and DBS in Advanced PD**.

**Symptom**	**Effect of levodopa**	**Effect of DBS**
Rigidity	Improves	Improves
Bradykinesia	Improves	Improves
Tremor	Improves	Improves
Gait/Balance	Individual components of gait and balance appear to respond uniquely	Evidence supports better results with GPi DBS
Dyskinesia	May worsen	Improves
Off time	Medicine adjustments may not improve off time in advanced PD	Improves
Cognitive Function	May worsen during off times	Studies have demonstrated worsened verbal fluency and set shifting abilities during complex tasks following STN DBS
Mood and apathy	May improve	May worsen with STN DBS, especially with rapid medication reduction and in patients with history of ICD
Speech	No change	May worsen, especially with bilateral STN DBS
Quality of life	Improves	Improves

*Key: PD, Parkinson's disease; DBS, deep brain stimulation; STN, subthalamic nucleus; GPi, internal segment of the globus pallidus; ICD, impulse control disorders*.

### Novel brain targets

To help address resistant axial and gait symptoms associated with PD, there has been persistent interest in pedunculopontine nucleus (PPN), a cholinergic nucleus located within the mesencephalic locomotor region (MLR), functionally defined as the area of the brainstem responsible for locomotion (Figure 2). Initial clinical investigations of PPN stimulation demonstrated improvement in axial and postural symptoms in PD patients with gait disturbances, although subsequent studies have tempered enthusiasm for this intervention with outcomes being highly variable (Mazzone et al., 2005; Plaha and Gill, 2005; Stefani et al., 2007; Ferraye et al., 2011; Thevathasan et al., 2012). Several critical issues have likely contributed to this variability, including disagreements over proper anatomic lead placement, whether PPN was utilized as a standalone target or combined with stimulation of other brain sites (e.g., along with STN stimulation), and unilateral vs. bilateral implantation.

Other locations of interest include the centromedian thalamus, zona incerta, and the substantia nigra (SNr). The SNr is also part of the MLR, with dense reciprocal interconnections to the PPN and brainstem circuitries. Early investigations have shown promise, demonstrating improvement in gait freezing with combined SNr/STN-DBS (Weiss et al., 2011, 2013) and gait and posture with SNr-DBS alone (Chastan et al., 2009). Larger and more rigorous studies are needed to determine the viability of these unique stimulation targets for refractory axial symptoms in PD. Clearly these are desired investigations as expansion of DBS to further address medically refractory symptoms of PD could once again revolutionize management of this disease.

### Novel lead design

**Figure 3:**
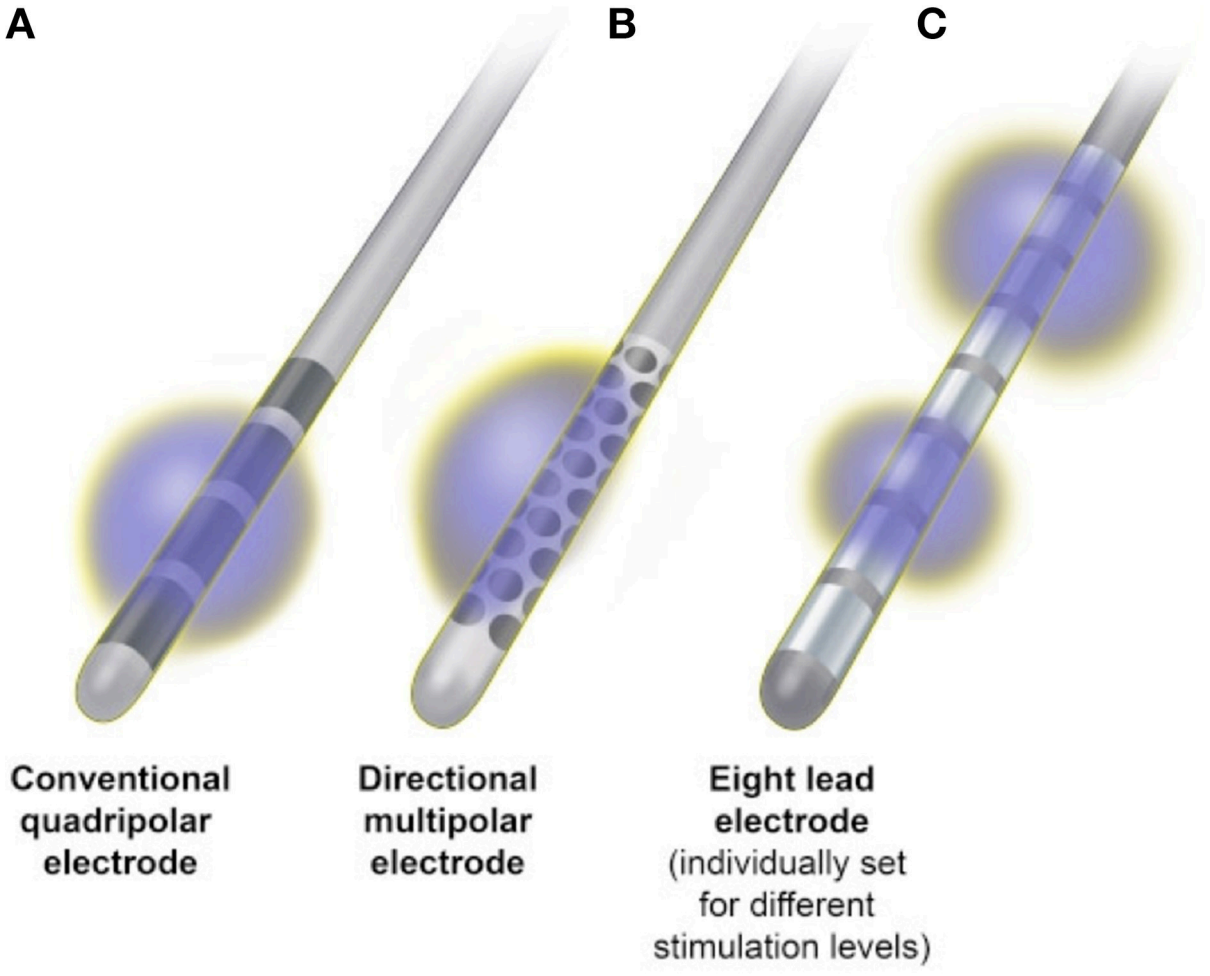
**Deep brain stimulation electrode configurations. (A)** Conventional quadripolar electrode producing a spherical electrical field that may spread outside the target area, causing side effects. **(B)** Multipolar 32 contact electrode that allows directional steering of the field, reducing the potential for stimulation side effects. **(C)** Eight contact electrode with multiple independent current control (MICC), enabling the allocation of completely different stimulation parameters independently to each electrode contact.

Since, the advent of therapeutic DBS more than 25 years ago, there has been little change to the classic DBS electrode, which consists of a flexible 1.27-mm- diameter cylinder with a stack of four platinum/iridium cylindrical contacts, 1 mm in height, spaced either 1 or 0.5 mm apart at the distal end (Medtronic Inc, Minneapolis, MN). The electrode is implanted into a brain target and attached to an implanted pulse generator (IPG), which provides stimulation as a continuous spherical electric field around the active contact(s). The electrical parameters for chronic stimulation are selected during DBS programming, the goal of which is to screen for the optimal electrode polarity, amplitude, pulse width, and frequency which avoids adverse effects of stimulation, while optimizing benefits and minimizing current consumption. At present, this is most commonly achieved by choosing either monopolar or bipolar stimulation and varying combinations of active contacts, thus adjusting current flow through the desired target area (Volkmann et al., 2006). In monopolar stimulation, the active contact is set as the negative pole, or cathode, and the internal pulse generator case is set as the positive pole, or anode. This creates a wide electric field with relatively equal spread of stimulation in all directions. In bipolar stimulation, another electrode contact serves as the anode, minimizing the spread of current and yielding a narrower area of stimulation (Butson and McIntyre, 2008) (Figure 3).

The optimal functional area within DBS target structures is limited in size and in close proximity to surrounding structures, thus classical monopolar or bipolar stimulation sometimes fails to provide sufficient beneficial effects or causes undesired side effects at stimulation amplitudes needed to sufficiently control symptoms. The emergence of stimulation-related adverse effects is an important limitation of DBS therapies, occurring regularly in up to 13% of patients (Timmerman et al., 2015). In fact, the current state-of-the-art lead design is particularly sensitive to surgical targeting errors that are tangential to the lead's primary axis and studies have demonstrated that targeting errors of only 2 mm can result in unwanted side effects that negate the intended therapeutic response (Montgomery, 2012). When conventional programming techniques fail to achieve desired results, interleaving stimulation (Medtronic Inc., Minneapolis, MN) may provide an alternative option. Interleaving is a novel and readily available feature that allows independent stimulation of two contacts of the quadripolar DBS electrode, which rapidly alternate from pulse to pulse between each other. Different values for voltage and pulse width may be used between the active contacts, which allow the clinician to shape the field of electrical stimulation along the vertical axis of the electrode. Initial investigations of this technology suggest that it may have limited utility in specific situations (Miocinovic et al., 2014).

To further address the limitations of available stimulation technology, segmented electrodes have been designed that allow better contouring of the electrical field, thus activating targeted neural pathways while reducing the risk of unwanted side effects. One such novel electrode design (Boston Scientific Corporation, Marlborough, MA) is capable of delivering multiple source constant current, enabling the allocation of completely different stimulation parameters independently to each of eight contacts of the electrode (Figure 3). A recently reported prospective, multicenter, non-randomized, open-label trial of bilateral STN DBS in 40 PD patients utilizing this technology demonstrated excellent motor benefit with improved quality of life measures (Timmerman et al., 2015). While this allows more tailored stimulation within the brain target, the current delivered will still encompass the tissue around the whole circumference of the active contact, limiting electrical field shaping to the vertical axis of the electrode.

Given the close proximity of subcortical DBS targets to surrounding neuronal structures and white matter pathways, there remains a need for true current steering and true electric field shaping around all three directional axes. Two approaches have been investigated to address this need, including a novel electrode with 32 contacts distributed evenly around the circumference of the lead (Figure 3), which enables current steering in four different directions (Contarino et al., 2014) and a lead with four rings, where each ring consists of three independent electrodes with three different orientations allowing independent stimulation in any of the three directions (Pollo et al., 2014). Investigations of both electrode designs have shown the ability to reproduce benefits equivalent to standard electrodes, while significantly widening the therapeutic window and positively influencing the thresholds for programming (Contarino et al., 2014; Pollo et al., 2014). Chronic implantation is now needed to establish the usefulness of directional steering in the long-term.

Further, advancements in DBS lead design continue with the goal of enhancing the spatial precision of stimulation (Connolly et al., 2016). With such progress, the possibility exists to target symptoms traditionally refractory to currently available neurostimulation algorithms, and to design new treatments, targeting small brain areas currently inaccessible because of commonly induced side effects. The indications for DBS continue to expand, and future targets for DBS could include small subnuclei in the hypothalamus, in the fornix, the upper brainstem, or even the medulla oblongata and spinal cord.

### Adaptable DBS

The therapeutic success of DBS depends not only on accurate surgical targeting and electrode implantation, but also on the ability to optimize stimulation parameters. Presently, DBS programming in the clinic is an iterative process in which stimulation settings are adjusted in order to maximize therapeutic benefits while minimizing side effects. Although many surgical patients require minimal stimulation adjustment following implantation, a significant number require several months of regular parameter modification before optimal therapeutic results are achieved (Okun et al., 2005; Kluger et al., 2011). A number of inherent limitations of DBS may contribute in these situations. While PD is a dynamic and progressive disease, DBS is classically delivered in an open-loop fashion, in other words, the neurostimulator is blind to the neural activity it is modulating and does not adjust its parameters based on any neural or kinematic feedback. This open-loop configuration means that any adjustment is dependent on the subjective experiences of both the patient and clinician; there is no objective feedback to support parameter optimization. In addition, the therapeutic response observed upon adjustment does not guarantee sustained therapeutic effects; disease progression, cognitive and motor load, mood, and concurrent drug therapy can all impact the effectiveness of stimulation, necessitating additional programming sessions (Obeso and Guridi, 2001; Kupsch et al., 2011). Further, DBS adjustment sessions are inefficient and time consuming, allowing only a limited exploration of settings during each procedure. Finally, programming can vary greatly depending on target location and the symptoms being treated for any given disorder (e.g., tremor may require different settings than gait).

To overcome these limitations and improve DBS delivery, the establishment of a “closed loop” system would allow therapy adjustment in real-time according to quantifiable and objective brain and behavioral changes while reducing the frequency of clinical interventions. A responsive DBS system capable of sensing local neuronal activity and automatically adjusting stimulation parameters in response to changes within the brain may improve clinical efficacy, decrease stimulation-induced side effects, and prolong battery life. Recent proof-of-principle studies have shown that by personalizing and optimizing stimulation parameters in real time, the efficacy and efficiency of DBS as an adaptable program can be potentially improved (Bronte-Stewart et al., 2009; Grahn et al., 2014). While individualized stimulation could revolutionize DBS delivery, a significant challenge is to determine a reliable and robust feedback signal in a chronically behaving human. Various responses have been considered and evaluated such as movement kinematics (Mera et al., 2011) or neurotransmitter fluctuations in a target nucleus (Grahn et al., 2014). However, perhaps the most thoroughly investigated biophysiological signal for an adaptive DBS system is the measurement of local neural activity.

The striatal dopamine depletion associated with PD produces strong neural synchronization, bursting and sustained low frequency oscillations within the basal ganglia motor circuit. In the unmedicated PD patient, local field potentials (LFPs) recorded from the STN show an exaggerated oscillatory activity in the beta range (13–30 Hz) while at rest. Resting state beta band power has the potential to be a useful metric to guide an adaptive DBS system, and has a number of potential advantages. Parkinsonian motor symptoms, including bradykinesia and rigidity, are related to strong and sustained activity in beta-frequencies (Kühn et al., 2006; Hammond et al., 2007; Little et al., 2012) while dopaminergic medication and high-frequency DBS attenuates abnormal beta activity (Brown, 2003; Bronte-Stewart et al., 2009; Whitmer et al., 2012). In addition, local field potentials can be directly recorded from the stimulating electrode allowing real time feedback and modulation. Early investigations utilizing beta band activity as a neural marker for an adaptive DBS system and have shown promise in improving stimulation efficiency (Little et al., 2013; Quinn et al., 2015). While clinical applications may be affected by variations between different PD phenotypes and susceptibility to artifact, further investigations into beta band as a reliable biophysiological signal for an adaptive DBS system are warranted and ongoing.

## Advances in imaging for deep brain stimulation

Along with new developments in the DBS technology itself, advances in imaging techniques and in related visualization software programs will provide a major impact on the field of invasive neurostimulation.

### Image guided DBS

The traditional method of DBS implantation employs detailed brain imaging to first delineate the intended target. This is typically achieved by use of stereotactic magnetic resonance imaging (MRI), which enables direct visualization of brain anatomy and the intended targets utilized for PD. Stereotaxy determines accurate localization of intracranial targets by ascertaining triplanar coordinates with reference to a fixed point, thus providing access to surgical targets deep within the brain in a minimally invasive fashion. The target is then constructed by use of graphic tools embedded within available stereotactic navigation software and registered in a stereotactic coordinate system. This planning stage allows pre-operative target adjustments based on individual patient anatomy and the selection of a skull entry point that avoids interrupting vessels of the cortical surface and sulci, ventricles, or other eloquent brain structures.

Functional neurosurgery involves the precise surgical targeting of anatomic structures to modulate neurologic function, such as abnormal movements. As such, accuracy and precision of electrode placement is of great importance. Most surgical teams utilize some form of electrophysiological mapping or stimulation testing to ensure that the optimal location within the target has been implanted. This exploration may consist of microelectrode recording of single neurons, local field potential recording of populations of neurons, or macrostimulation of the intended target with direct observation of the patient for improvement in symptoms and/or stimulation-induced side effects. Microelectrode recording provides adjustment for interpatient variability in physiology and symptomatology, and recording during passive movement of a limb can confirm an association with motor function and help ensure implantation within the motor subterritories of the intended brain target. However, these techniques require that the patient be awake for much of the procedure, which is difficult for some to tolerate. In addition it is time consuming, technically demanding, and often necessitates multiple brain penetrations.

Electrophysiological mapping helps ensure proper anatomic position of the DBS electrode in the brain, which is essential for safety, selection of stimulation parameters, and the ultimate success of the therapy. Various types of imaging modalities may also aid surgical targeting and both computed tomography (CT) and MRI have been successfully integrated into the intraoperative workflow of DBS surgery. One approach involves imaging the implanted microelectrode with registration back to the pre-operative planned trajectory. The physiologic data are then interpreted in light of the position of the electrodes on the scan. A subsequent track can be created, or the DBS lead can replace one of the microelectrodes. Imaging of the DBS lead after placement assures the surgeon that its position is acceptable. Evidence supports that this approach allows for correction of the average 2-mm targeting error seen with traditional stereotactic approaches (Starr et al., 1999; Kelman et al., 2010; Shahlaie et al., 2011; Holloway and Docef, 2013).

For nuclei targeted for PD, such as the GPi and STN, which are visible on particular MRI sequences, new techniques have been developed for implanting DBS leads using real-time interventional MRI (iMRI) guidance without the use of microelectrode recordings or intraoperative test stimulation (Martin et al., 2009; Starr et al., 2009, 2010). By utilizing a skull-mounted aiming device (SmartFrame; MRI Interventions, Irvine, CA) in collaboration with a software platform (ClearPoint; MRI Interventions), this system can stream MR images in real time from any 1.5-T or 3-T scanner, and guide the surgical team through the implantation procedure. DBS implantation is performed entirely within the MRI scanner, and relies on direct visualization of the STN or GPi as the sole method of targeting. A stereotactic frame is not needed, MRI is the only imaging modality used, so no image fusion is needed and targeting is performed after cranial opening so brain shift can be taken into account.

This method marks a departure from traditional stereotactic techniques by eliminating the need for preoperative MR images, which cannot account for intraoperative brain shift due to pneumocephalus or CSF loss. Real time imaging enhances the detection of intraoperative complications and reduces the procedure to a single brain penetration in most cases (Starr et al., 2010; Larson et al., 2012). This technique may also be faster with a higher accuracy than a stereotactic frame surgery, while producing clinical outcomes that are comparable to traditional, awake procedures (Ostrem et al., 2013, 2016). Using the ClearPoint system, clinically acceptable placement can be obtained with a single brain penetration in 98% of cases, with an average application accuracy of 0.6 to 0.8 mm from the intended target (Ostrem et al., 2013, 2016). Finally, surgery can be performed under general anesthesia, and does not require withholding of parkinsonian medications, thus broaden the accessibility to those who might not otherwise be able to tolerate awake surgery.

### Advanced imaging techniques

The basal ganglia are components of segregated, largely closed, reentrant loop circuits that originate in the cerebral cortex, traverse the basal ganglia and thalamus, and return to their individual sites of origin (Wichmann and Delong, 2006). PD results from dysfunction of multiple motor and nonmotor neural circuits. Although the clinical efficacy of DBS for the cardinal features of PD has been demonstrated, the exact mechanisms by which it induces these effects are still not well understood. Since DBS motor outcomes are similar to basal ganglia structural lesioning procedures, it was originally proposed that high frequency electrical stimulation induced inhibition by depolarization blockade of abnormal neuronal firing within the dysfunctional motor circuit (Beurrier et al., 2001). However, DBS mechanisms appear to be quite complex, activating axons while simultaneously inhibiting soma (Grill and Mclntyre, 2001; Vitek, 2002; McIntyre et al., 2004; McIntyre and Hahn, 2010). While the net benefit of neurostimulation would be the sum of its effects on local and remote structures, recent research has posited that DBS relieves symptoms by the selective stimulation of white-matter tracts (Henderson, 2012) or by the normalization of pathologic global functional networks (Kringelbach et al., 2011).

While much of the current understanding of basal ganglia structure and function originated from animal studies or human lesion or post mortem studies, white matter pathways have recently been imaged *in vivo* in humans using diffusor tensor imaging (DTI). Such advanced neuroimaging techniques have helped validate many components of these circuits, while also refining some overlap between pathways and enhancing information about smaller subregions (Weingarten et al., 2015). Beyond improving the understanding of network structure, DTI and fiber tracking have been used to visualize white matter tracts as a target area. Enhanced visualization of deep brain white matter tracts raises the possibility of improved anatomically guided DBS, which would obviate the need for patients to be awake, could expedite the placement of the DBS leads, reduce the need for repositioning, decrease operating time, and improve overall patient experience and outcome. Further, detailed circuit mapping could allow targeting of traditionally refractory PD symptoms (e.g., gait freezing) or avoidance of common side effects (e.g., speech changes).

Accurate electrode placement is paramount for maximizing therapeutic benefits while minimizing potential side effects and this begins with visualization of the DBS target during surgical planning. As DBS acts predominantly on axons and dendrites nearest the electrode, neuroimaging techniques that improve approximation to intended and pathogenic targets may dramatically improve current surgical outcomes and overcome limitations. High-quality, distortion-free images are essential for this endeavor. Despite advances in MRI technology, challenges still remain with currently utilized 1.5- and 3-T T2 MRI sequences, including geometrical distortion and poor image quality. Newer MRI sequences, including susceptibility-weighted imaging (SWI) and T2-weighted magnitude imaging (T2^*^WI), as well as image reconstruction methods such as susceptibility-weighted phase imaging (SWPI) and quantitative susceptibility mapping (QSM), have shown promise in improving target contrast and better defining target borders (Chandran et al., 2015; He et al., 2015).

Functional connectivity studies utilizing fMRI and multimodel analysis have demonstrated widespread changes in PD patients, including alterations to motor networks (Weingarten et al., 2015). Further, evidence supports an overlap between deficits in anatomical and functional connectivity within the sensorimotor circuit in PD, indicating a possible link between brain structure and function (Sharman et al., 2013). As DBS has been shown to modulate all the major components of the motor circuit (Kahan et al., 2014), utilization of network analyses in DBS planning and programming may allow individual tailoring of therapy based on unique features of the abnormal network.

Future advances and refinements in neuroimaging are likely to add to the complex neurodegenerative picture of PD that has already developed. However, with more individualized neuroimaging comes the potential for personalized approaches for surgical treatment such as tailored DBS based on symptoms, potential for progression, and co-morbidities.

In all, the DBS procedure continues to be refined, with the goal of reducing errors and side effects, while improving patient comfort and optimizing successful outcomes.

## Conclusions and future directions

DBS was pioneered nearly three decades ago and its benefits have been repeatedly demonstrated in patients with medically intractable PD. Such robust outcomes have lead great interest in the use of DBS in the treatment of other neurological conditions such as chronic pain and medically refractory psychiatric/behavioral conditions such as obsessive-compulsive disorder or depression. In fact, the neurostimulation has been explored in more than 40 brain sites for 30 clinical disorders (Hariz et al., 2013). In addition to its therapeutic benefits, DBS proved to be a highly valuable tool for research, providing a minimally invasive probe for stimulation and recording of electrical signals from the implanted targets. This research has provided new insights into brain network function and dysfunction in neurologic and psychiatric disorders.

To accommodate the rapid expansion in scope, novel technologies are under development to provide clinicians with new tools making targeting, programming and overall management easier. These technologies vary in focus though converge toward providing safer and more accurate placement of electrodes with a subsequent optimization of therapeutic benefit. Careful evaluation of emerging technology will be imperative to demonstrate any usefulness and/or advantage over what is available today.

## Author contributions

All authors listed, have made substantial, direct and intellectual contribution to the work, and approved it for publication.

### Conflict of interest statement

PH is the director of the Duke University Parkinson Disease and Movement Disorders fellowship, which receives grant support from Medtronic. MS works for Duke University, and has received consultancy fees from Eli Lilly, Merz, Osmotica, Pfizer, SK Life Sciences, Allergan, Avid, Best Doctors, Biotie, Lundbeck, Neuronova, Novartis Pharma (Japan), Saraepta Therapeutics, and Sunovion Pharmaceutics, Inc. Dr. MS has also received grants from the Michael J. Fox Foundation, the NIH, the Parkinson Study Group, and Pharma 2B, royalties from Informa Press for the Handbook of Dystonia and Duke University for the Wearing Off Questionnaire. He has also received payment for development of educational presentations from the University of Kansas, the University of Miami, and the University of Rochester. Dr. MS also received paid travel accommodations from the Cleveland Clinic Neurological Institute, the Movement Disorder Society, and the National Parkinson Foundation.
